# Improving the Water Resistance and Mechanical Properties of Feather Keratin/Polyvinyl Alcohol/Tris(Hydroxymethyl)Aminomethane Blend Films by Cross-Linking with Transglutaminase, CaCl_2_, and Genipin

**DOI:** 10.3390/ma11112203

**Published:** 2018-11-07

**Authors:** Shufang Wu, Xunjun Chen, Minghao Yi, Jianfang Ge, Guoqiang Yin, Xinming Li

**Affiliations:** 1 Green Chemical Engineering Institute, Zhongkai University of Agriculture and Engineering, Guangzhou 510225, China; SFWu2018@163.com (S.W.); MHYi0848@163.com (M.Y.); ge650704@163.com (J.G.); yingq007@163.com (G.Y.); lixinming@sina.com (X.L.); 2 Guangzhou Key Laboratory for Efficient Utilization of Agricultural Chemicals, Guangzhou 510225, China

**Keywords:** blend film, feather keratin, cross-linking, water resistance, mechanical properties

## Abstract

The high moisture sensitivity of feather keratin/polyvinyl alcohol/tris(hydroxymethyl)aminomethane (FK/PVA/Tris) blend films hinders their application in the packaging field. Thus, in order to improve the water resistance and mechanical properties of such blend films, we attempted cross-linking the blend film with cross-linking agents such as transglutaminase (TG), CaCl_2_, and genipin. Obvious differences in the morphology of the blended films were observed by scanning electron microscopy before and after cross-linking, indicating that cross-linking can inhibit the phase separation of the blend film. Conformational changes in the blend films after cross-linking were detected by Fourier transform infrared spectroscopy. Importantly, from examination of the total soluble mass, contact angle measurements, and water vapor permeability tests, it was apparent that cross-linking greatly improved the water resistance of the blend films, in addition to enhancing the mechanical properties (i.e., tensile strength and elongation at break). However, cross-linking was also found to reduce the oxygen barrier properties of the blend films. Therefore, cross-linking appears to be an effective method for promoting the application of FK/PVA/Tris blend films in the packaging field.

## 1. Introduction

Keratin, a natural biopolymer, is found in abundance (80–90% protein content) in animal hair, feathers, and hooves. Indeed, feathers and hairs are derivatives of the keratinization of animal epidermal cells. The extraction of keratin can be accomplished by physical methods (such as heating or pressurization [1]), chemical methods [2] (including oxidation [3], reduction [3,4,5], sulfitolysis [6], ionic liquids [7], and alkali hydrolysis [8]), and microbial degradation methods [9,10]. It is estimated that feathers, feather poles, and scraps from livestock slaughtering (or their by-products) amount to more than one million tons per year [11,12], with the majority of this being disposed of as waste. This not only causes environmental pollution, but also wastes resources. Thus, if keratin can be used to prepare packaging films via a suitable process (viz., hot pressing [13], solvent-casting [14,15,16,17], electrospinning [18,19,20], compression molding [21], and layer-by-layer (LbL) deposition [22]), the white pollution issue caused by the use of synthesized polymer materials in the packaging field can be reduced, in addition to improving the economic benefits of keratin.

Although keratin is readily available, biodegradable, renewable, and inexpensive, its application in packaging is limited due to its brittle nature and poor toughness. However, these issues can be addressed through blend modification. More specifically, we previously found that blending feather keratin (FK) with polyvinyl alcohol (PVA) and tris(hydroxymethyl)aminomethane (Tris) improved the mechanical properties and oxygen barrier properties of the protein films [23]. However, the moisture sensitivities of such blend films were increased, although modification through cross-linking was found to reduce the moisture sensitivities of the blend films. In this context, commonly employed chemical cross-linking agents include aldehydes such as formaldehyde [24,25], glyoxal [26,27,28], glutaraldehyde [29], cinnamaldehyde [30], and dialdehyde starch [16]. These aldehydes react with the free amino groups in the protein to form a cross-linked network structure. However, as the majority of aldehydes are toxic and can be released into the environment during use, the use of non-toxic and environmentally friendly cross-linkers for the modification of proteins have attracted significant interest.

For example, transglutaminase (TG) is one such environmentally friendly cross-linker. TG is an enzyme that catalyzes acyl transfer reactions. In such reactions, the γ-carboxyamide groups of peptide-bound glutaminyl residues are the acyl donors. The primary amino group acts as an acyl acceptor, which binds to the γ-carboxamide group of the glutaminyl residue to form a monosubstituted γ-amide of glutamic acid. Therefore, this combination of an amino compound with a glutamic acid residue produces intramolecular and intermolecular cross-links. Indeed, it has been reported that the use of TG cross-linked whey protein [31], casein [32,33], keratin [32,33], and soy protein isolate [32,33] has improved the mechanical properties of the resulting protein films and reduced their water sensitivities.

CaCl_2_ is also considered an environmentally friendly cross-linker. In this case, Ca^2+^ forms a network structure through ionic bonding with the protein carboxylic groups [34]. It has been reported that the addition of formaldehyde and CaCl_2_ to whey protein film enhances the mechanical properties and insolubility behavior, in addition to increasing the glass transition temperature [25]. In addition, Arabestani et al. [34] found that CaCl_2_ could improve the barrier properties and hydrophobicity of the protein film, which was cast from bitter vetch protein concentrate (BPC) and glycerol.

Genipin is also a commonly used and effective natural cross-linking agent. It is found in traditional Chinese medicine, and is mainly extracted from gardenia fruit, which can spontaneously react with primary amine groups to form a dark blue pigment [35]. The probable reaction mechanisms between primary amine groups and genipin include two reaction processes [36]. The first step is the nucleophilic attack of the genipin olefin carbon atom at C3 by a primary amino group to form a heterocyclic compound of genipin cross-linked with a primary amine group [37]. The second reaction is the nucleophilic substitution of the ester group possessed by genipin to form a secondary amide bond [38]. Motta et al. [39] reported that the use of genipin cross-links stabilized the silk fibroin/silk protein film and improved the mechanical properties of the blend films. In addition, You et al. [40] found that the use of genipin to cross-link silk protein effectively reduced the degradation rate of the films, while Vasconcelos et al. [41] reported that novel silk fibroin/elastin scaffolds exhibited higher thermal stabilities, a pH-swelling dependence, and reduced biological degradation and drug release rates following genipin cross-linking. Furthermore, Bigi et al. [42] found that gelatin films prepared by cross-linking with genipin exhibited reduced swelling in physiological solutions, in addition to enhancing the film thermal stabilities.

However, to the best of our knowledge, the use of such reagents for the cross-linking of FK is rare. Thus, we herein report the use of TG, CaCl_2_, and genipin to cross-link FK/PVA/Tris blend films. Ultimately, we wish to investigate the influence of these three cross-linking agents on the properties of cast FK/PVA/Tris blend films.

## 2. Materials and Methods

### 2.1. Materials

Polyvinyl alcohol (analytical grade, degree of polymerization = 1700, degree of alcoholysis = 99) was obtained from Aladdin Ltd. (Shanghai, China). Tris(hydroxymethyl)aminomethane (analytical grade) was purchased from the Shanghai Ebene Chemical Reagent Co., Ltd. (Shanghai, China). TG (food grade) was purchased from Shanghai Xiangrui Biotechnology Co., Ltd. (Shanghai, China). Anhydrous CaCl_2_ (analytical grade) was obtained from Tianjin Baishi Chemical Co., Ltd. (Tianjin, China). Genipin (content >98%, chromatographically pure), was purchased from Zhixin Biotechnology Co., Ltd. (Fuzhou, China). The chicken feather keratin (FK) powder was prepared as described in our previous work [23].

### 2.2. Preparation of the 0.1% Cross-Linking Agent Solutions

The TG and CaCl_2_ solutions were prepared by adding TG and CaCl_2_ powder (0.1 g each) to deionized water (99.9 g) at 25 °C with continuous stirring over 5 min. The genipin solution was prepared by adding genipin powder (0.1 g) to deionized water (99.9 g) with continuous stirring at 40 °C for 60 min.

### 2.3. Preparation of the Blend Films

As described in our previous work [23], P-40-25 (FK:PVA weight ratio of 60:40, and a Tris content of 25 wt % relative to the total weight of FK and PVA) was selected as the control group, and the cross-linking agent was added on the basis of P-40-25 for cross-linking modification. More specifically, the FK/cross-linker solution was prepared by adding FK (1.2 g) to the 6% Tris solution (8.33 g) (total weight of FK and PVA was 2 g), and adding the 0.1% cross-linker solution (1 g, 2 g, 6 g, or 10 g, accounting for the total weight of FK and PVA, 0.05%, 0.1%, 0.3%, or 0.5%) with continuous stirring at 40 °C for 30 min. Mixtures of the FK/cross-linker solution and the 6% PVA aqueous solution (13.33 g) were prepared as film-forming solutions with continuous stirring at 40 °C over 2 h. Specific amounts of each component are outlined in Table 1. The obtained solutions were poured onto polypropylene dishes (15 × 18 cm) and dried in a humidity chamber at 25 °C and 50% relative humidity for 24 h, after which time the desired blend films were easily removed from the dishes by peeling. The series of prepared films were coded as x-TG, x-CaCl_2_, and x-genipin, where x is the weight percentage of cross-linker relative to the total weight of FK and PVA in the film. The obtained films were conditioned at 25 °C and 50% relative humidity for 48 h prior to testing.

### 2.4. Characterization

The cross-sectional was prepared by freezing the film samples in liquid nitrogen, and the surface morphologies of the prepared film samples were observed by scanning electron microscopy (SEM, EVO 18; Carl Zeiss, Jena, Germany) at a 15-kV accelerating voltage. Prior to the SEM observations, the samples were coated with a fine gold layer for 45 s.

Fourier transform infrared (FTIR) spectroscopy of the prepared film samples was measured with an infrared spectrometer (Spectrum 100, Perkin-Elmer, Fremont, CA, USA) using the attenuated total reflectance (ATR) mode in the wave number range from 650 cm^−1^ to 4000 cm^−1^ (eight scans per wavenumber). The obtained spectra were analyzed using Omnic software (OMNIC 8.2, Thermo Fisher Scientific-CN, Shanghai, China).

The mechanical properties of the prepared film samples, including tensile strength and elongation at break, were carried out with a microcomputer-controlled electronic universal testing machine (CMT6503, Shenzhen MTS Test Machine Company Ltd., Shenzhen, China). The film samples were cut into a 10 mm × 75 mm size, and the thickness of the samples were measured with a digital external micrometer (accurate to 0.001 mm). A speed of 10 mm/min, a fixture distance of 40 mm, and a load of 100 N were used in the experiment, according to the ASTM D 822 standard. The measurements were performed in triplicate, and average values were calculated.

The total soluble mass (TSM) of the prepared film samples in water at 25 °C were measured according to the procedure reported by Dou et al. [16] with slight modifications. Initially, the square specimens (40 mm × 40 mm) were preconditioned by drying in an air oven at 70 °C for 24 h, prior to their removal from the oven, cooling to room temperature, and immediate weighing (*W*1). The preconditioned specimens were then immersed in a beaker containing deionized water (100 mL), sealed with plastic wrap, and placed in a humidity chamber at 25 °C for 24 h. The resulting wet specimens were dried once again in an oven at 70 °C for 24 h; then, they were removed, cooled to room temperature, and then immediately weighed again (*W*2). The TSM values of the films were calculated using the following equation:

TSM (%) = (*W*1 − *W*2)/*W*1 × 100% (1)

All of the measurements were conducted in triplicate, and average values were calculated.

Contact angle measurements were measured with an automatic contact angle meter (Theta, Biolin Scientific Ltd., Espoo, Finland). More specifically, the prepared film samples were attached to a glass slide, laid flat on a stage, and then deionized water droplets (3–7 μL) were extruded from the needle tube onto the sample surface. The contact angle was evaluated as the average of the measurements on both sides of the water droplet.

The water vapor permeability (WVP) values of the prepared film samples were carried out with a water vapor transmittance tester (W3/030, Labthink Ltd., Jinan, China). The test temperature was 38 °C, the relative humidity was 90%, and the weighing interval was 2 h. All of the tested samples were cut into circles with radius of 1.5 cm. The measurements were carried out in triplicate, and average values were calculated.

At last, the oxygen permeability (OP) values of the prepared film samples were performed with an oxygen permeability tester (VAC-VBS, Labthink Ltd., Jinan, China). The test gas pressure of 1.01 × 10^5^ Pa and upper and lower degassing times of 4 h were used in the experiment, according to the GB/T 1038-2000 standard. All of the tested samples were cut into circles with radii of 2.75 cm. The measurements were carried out in triplicate, and average values were calculated.

### 2.5. Statistical Analysis

Data were expressed as mean ± standard deviation (mean ± SD). Statistics analysis were performed using one-way analysis of variance (ANOVA) with SPSS Statistics software (Version 17.0, Spss-China Co., Shanghai, China). Differences of means were processed by Duncan’s multiple range test, and significance was defined as *p* < 0.05.

## 3. Results and Discussion

### 3.1. Examination of the Film Morphologies

Upon visual examination, all of the films were found to be macroscopically uniform and optically transparent. In addition, the surfaces of the blend films appeared smooth and without any obvious cracks or pores. All of the films were yellowish in color, with the exception of the genipin cross-linked films, which changed from yellow-green to blue-green to dark blue upon increasing the genipin content. The film appearances did not change with an increase in the content of the cross-linking agent.

**Figure 1:**
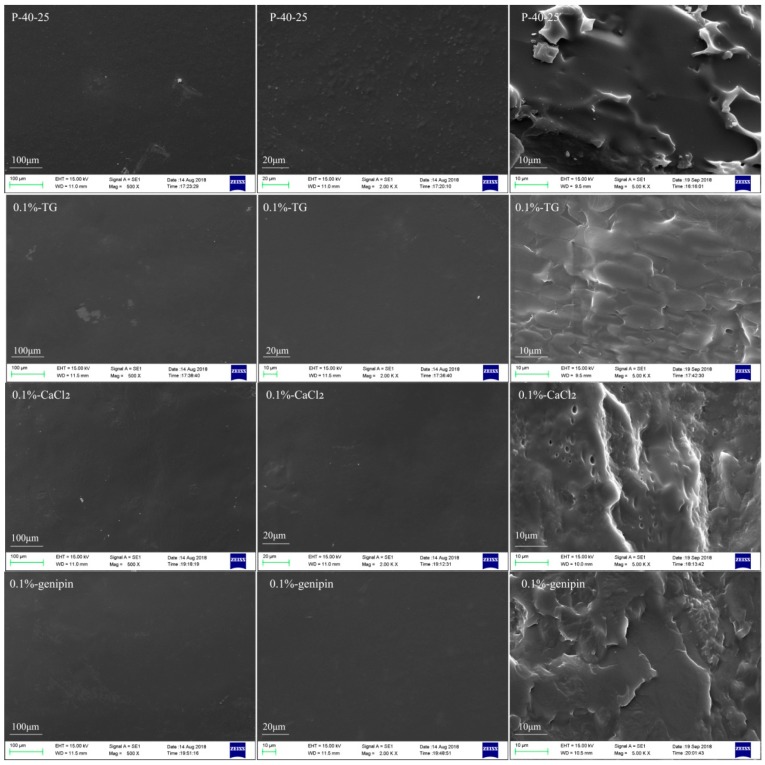
Representative SEM images revealing the surface morphologies (**left**, 500×; **center** 2000×) and the fracture morphologies (**right**, 5000×) of the blend films.

SEM observations were then carried out to obtain a better insight into the prepared film homogeneity and microstructure. SEM images of the surfaces of selected blend films (i.e., P-40-25, 0.1%-TG, 0.1%-CaCl_2_, and 0.1%-genipin) are presented in Figure 1. As shown, the surface of P-40-25 was continuous and no pores were observed, although teardrop-shaped bulges were observed at higher magnifications, which can be assigned to the different agglutination kinetics of FK and PVA during the dry film formation process [16]. Upon the cross-linking of P-40-25 with the three different cross-linking agents, the film surfaces appeared smoother than that of P-40-25, and no obvious teardrop-shaped bulges were observed at higher magnifications. The uniformity of the blended films could be more accurately judged by observing their fracture morphologies. Specifically, P-40-25 exhibited a rough fracture surface with a few small particles inside the matrix, suggesting that phase separation took place. Indeed, such phase separation was previously observed in FK/PVA blend films [16] and in FK/sodium alginate blend films [17]. In contrast, the fracture surface of the cross-linked films did not display any particles, and the more uniform surface were observed; this was likely due to chemical cross-linking, which effectively inhibited the phase separation of the blend film. However, a number of cross-linked agglomerates were observed in the form of spherical particles in our FK/PVA/Tris blend films cross-linked with TG, CaCl_2_, and genipin, as reported previously for FK/PVA blend films cross-linked with dialdehyde starch [16]. Therefore, it was apparent from the SEM results that a phase separation occurred in the FK/PVA/Tris blend system, and that this could be suppressed by the addition of cross-linking agents.

### 3.2. FTIR Analysis

**Figure 2:**
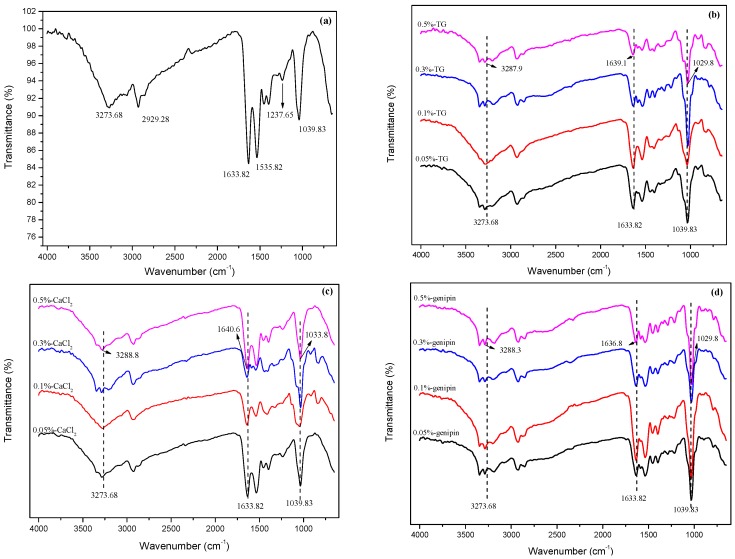
Fourier transform infrared (FTIR) spectra of (**a**) P-40-25, (**b**) P-40-25 cross-linked by TG, (**c**) P-40-25 cross-linked by CaCl_2_, and (**d**) P-40-25 cross-linked by genipin.

The FTIR spectra that was recorded of P-40-25 is shown in Figure 2a, where the signals observed at 3327.68 cm^−1^, 2929.28 cm^−1^, and 1633.82 cm^−1^ were attributed to the O–H and N–H association peaks, the –CH stretching vibration peak, and the characteristic peak of the amide I band (C=O stretching vibration), respectively. Furthermore, the peaks at 1535.82 cm^−1^, 1237.65 cm^−1^, and 1039.83 cm^−1^ corresponded to the characteristic absorption peak of the amide II band (the N–H bending vibration), the amide III band (the C=O bending and C–N stretching vibrations), and the C–O stretching vibration peak, respectively. In addition, Figure 2b–d show the FTIR spectra recorded for P-40-25 cross-linked by TG, CaCl_2_, and genipin, respectively, where significant changes were observed from the spectrum obtained for P-40-25 alone. It was observed that upon increasing the cross-linker concentration, greater shifts in the peak positions were observed. Specifically, the characteristic signals of P-40-25 at 3327.68 cm^−1^, 1633.82 cm^−1^, and 1039.83 cm^−1^ were shifted to 3287.9 cm^−1^, 1639.1 cm^−1^, and 1029.8 cm^−1^ for 0.5%-TG (corresponding to O–H and N–H association peaks, the amide I band, and the C–O stretching vibration absorption peak, respectively). Similarly, these peaks were shifted to 3288.8 cm^−1^, 1640.6 cm^−1^, and 1033.8 cm^−1^ for 0.5%-CaCl_2_, and to 3288.3 cm^−1^, 1636.8 cm^−1^, and 1029.8 cm^−1^ for 0.5%-genipin. These changes indicate that the addition of these cross-linking agents changed the conformation of the blend film, which was likely due to the formation of new chemical bonds.

To determine the effect of the cross-linking agents on the conformational changes of FK in the composite film, the amide I region (1600–1700 cm^−1^) was examined, where the broad bands situated at 1610–1640 cm^−1^ can be attributed to β-sheets, those at 1640–1650 cm^−1^ can be assigned to random coils, those at 1650–1660 cm^−1^ correspond to α-helices, and those at 1660–1700 cm^−1^ can be attributed to β-turns [33,43]. Thus, the secondary structures were expressed as a percentage of corresponding area by ratio to the total amide I band area, and the results are outlined in Table 2. As indicated, the α-helix content increased for the cross-linked films. According to the results of Wu et al. [33], greater proportions of α-helices improve the stability of a protein, thereby suggesting that the addition of such cross-linking agents improves the stability of the blend film. In addition, the β-sheet content was reduced, which indicates that the orderly structure of the protein was reduced [44], while the β-turn and random curl contents increased, which was likely related to the weak forces imparted by the cross-linking agents.

### 3.3. Mechanical Properties

**Figure 3:**
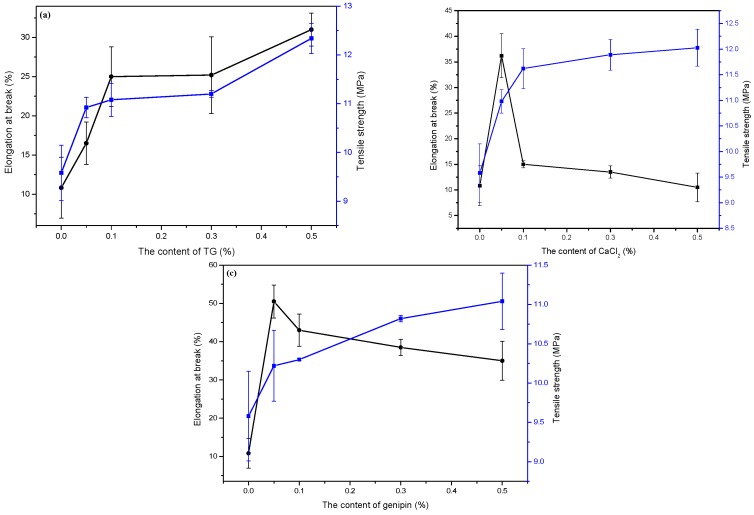
Tensile properties of (**a**) P-40-25 cross-linked by TG, (**b**) P-40-25 cross-linked by CaCl_2_, and (**c**) P-40-25 cross-linked by genipin.

As shown in Figure 3, the addition of the three cross-linking agents improved the mechanical properties of the FK/PVA/Tris blend films, as the elongation at break and the tensile strength were found to increase compared with those of the control film. More specifically, the elongation at break increased from 10.83% (P-40-25) [23] to 31%, 36.2%, and 50.5% (corresponding to TG, CaCl_2_, and genipin cross-linking, respectively), while the tensile strength increased from 9.58 MPa (P-40-25) [23] to 12.34 MPa, 12.03 MPa, and 11.04 MPa (corresponding TG, CaCl_2_, and genipin cross-linking, respectively). Similarly, Wu et al. [33] reported that the mechanical properties of type I collagen were improved upon cross-linking with casein, keratin, and soy protein isolate using TG. In addition, Avena et al. [45] reported that the mechanical properties of a protein-based edible film were improved through Ca^2+^ cross-linking, while Motta et al. [39] reported that the mechanical properties of silk fibroin/silk fibroin composite films with genipin-induced cross-linking were superior to those of untreated films.

However, it is worth noting that the three different cross-linking agents imparted different effects on the mechanical properties of FK/PVA/Tris blend films, and these mechanical properties varied with different concentrations of the various agents. For the composite film with TG cross-linking, the tensile strength and elongation at break increased upon increasing the TG content. This can be accounted for by the TG reacting with the amino groups in FK and Tris to form a three-dimensional network structure and improve the mechanical properties of the composite film. For the composite films with CaCl_2_ and genipin cross-linking, the tensile strength increased with increasing cross-linking agent concentrations, while the elongation at break initially increased prior to decreasing at higher concentrations. This phenomenon may be due to CaCl_2_ and genipin producing a three-dimensional network structure with FK, thereby reducing structural defects in the blend films. At higher concentrations of CaCl_2_ and genipin, the three-dimensional network structure is tighter due to additional cross-linking, which is not conducive to the sliding of the molecular chain, thereby resulting in a decrease in the elongation at break.

### 3.4. Moisture Sensitivities of the Blend Films

#### 3.4.1. Total Soluble Masses of the Blend Films

**Figure 4:**
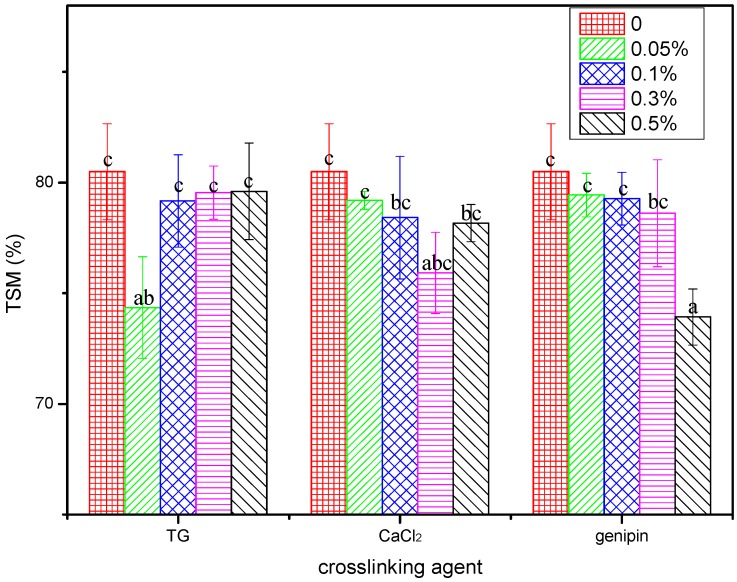
The total soluble masses of the blend films. Different lowercase letters indicate significant differences (*p* < 0.05).

The stability of a protein-based film in water can be measured in the context of the total soluble mass (TSM). In general, protein-based films are highly sensitive to moisture, and so are unstable in water. Therefore, cross-linking is the most common and useful method to reduce the TSM of protein-based films in water [46,47,48]. Thus, the TSM values obtained for the cross-linked FK/PVA/Tris blended films in water at 25 °C are shown in Figure 4, where it is apparent that the three cross-linking agents lowered the TSM values of the FK/PVA/Tris cross-linked films compared to that of P-40-25, thereby confirming a reduction in the moisture sensitivities of the films and increased stabilities.

In addition, we found that the optimal cross-linking concentration differed between the three cross-linking agents. More specifically, for the blend film cross-linked by TG, a TG content of 0.05% resulted in the TSM of the cross-linked film, reaching a minimum of 74.35%. Upon increasing the TG content, the TSM value increased slightly, but was lower than that of the control film, thereby suggesting that a TG content of 0.05% resulted in the maximum degree of cross-linking. Further increases in the water-soluble TG resulted in an increase in the TSM due to its dissolution.

In addition, for the blend film cross-linked by CaCl_2_, the TSM decreased upon increasing the CaCl_2_ content, with a minimum of 75.92% being reached with a CaCl_2_ content of 0.3%. Similar results to those described for TG were observed upon increasing the CaCl_2_ content further.

Furthermore, for the blend film cross-linked by genipin, again, the TSM decreased upon increasing the genipin content. In this case, with a genipin content of 0.5%, the TSM of the cross-linked film reached a minimum of 73.93%. However, it should be noted that the blend films broke up into smaller fragments during the dissolution process, which made collection and weighing difficult, and so the measured data was biased.

#### 3.4.2. Contact Angles of the Blend Films

**Figure 5:**
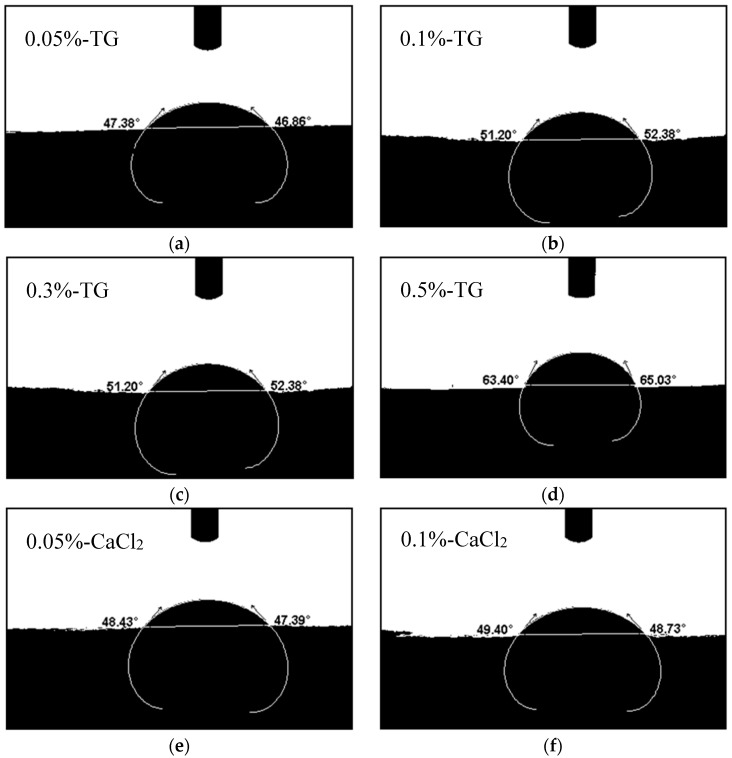

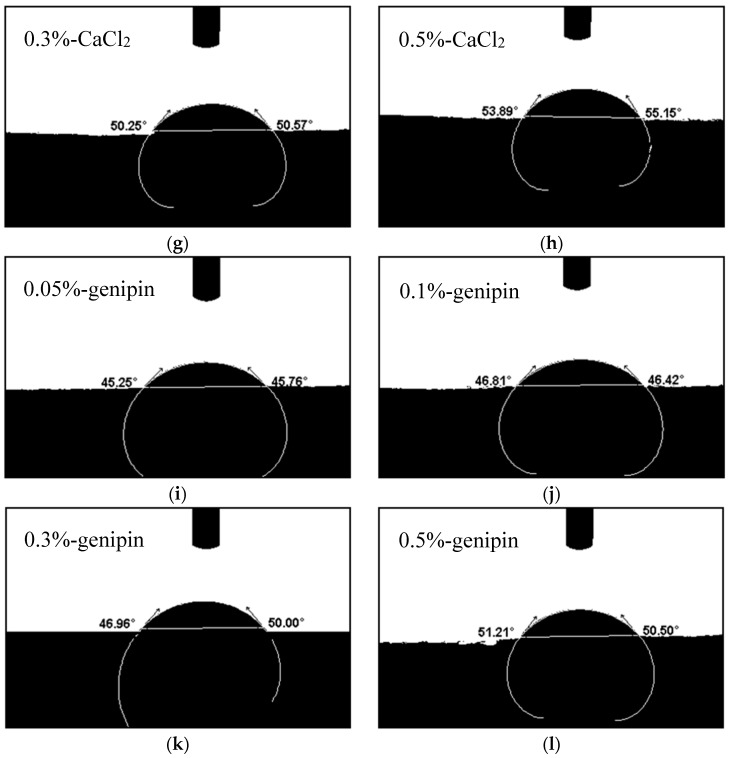
The contact angles for the various blend films: (**a**) 0.05%-TG, (**b**) 0.1%-TG, (**c**) 0.3%-TG, (**d**) 0.5%-TG, (**e**) 0.05%-CaCl_2_, (**f)** 0.1%-CaCl_2_, (**g**) 0.3%-CaCl_2_, (**h**) 0.5%-CaCl_2_, (**i**) 0.05%-genipin, (**j**) 0.1%-genipin, (**k**) 0.3%-genipin, and (**l**) 0.5%-genipin.

To judge the effect of the three cross-linkers on film hydrophilicity, we investigated the contact angles of P-40-25 containing various TG, CaCl_2_, and genipin contents. The contact angle photo of each sample is shown in Figure 5, and the contact angles increased gradually upon increasing the contents of the cross-linking agents. More specifically, when the cross-linking agent content was 0.5%, the contact angles reached their maximum values of 64.22°, 54.52°, and 50.86° for cross-linking by TG, CaCl_2_, and genipin, respectively. Compared with P-40-25, whose contact angle was 43.56° [23], increases of 10°–20° were observed, indicating that the cross-linkers effectively reduced the moisture sensitivity of the composite film; this was likely due to the formation of a three-dimensional network structure. Furthermore, the cross-linking reaction may have had an effect on the orientation of the hydrophobic groups toward the surface of the films, which in turn improved the water resistance of the FK/PVA/Tris composite films.

### 3.5. Barrier Properties of the Blend Films

The barrier properties of blend films directly affect their use in the packaging field. Therefore, it is necessary to study the barrier properties of the blend films. To determine the effect of the three cross-linkers on film barrier properties, we investigated the water vapor permeability (WVP) and the oxygen permeability (OP) of P-40-25 containing various TG, CaCl_2_, and genipin contents.

#### 3.5.1. Water Vapor Permeability (WVP)

The effects of the three cross-linking agents on the WVP values of P-40-25 are outlined in Table 3, where it is apparent that the addition of these cross-linking agents reduced the WVPs of the FK/PVA/Tris composite films compared to that of the control film. This was consistent with the TSM data. Similarly, Avena et al. [45] reported that the WVP of a protein-based edible film was significantly reduced by adding Ca^2+^, while Dou et al. [15] reported that a cross-linked network was formed in the FK/PVA composite films by the addition of dialdehyde starch, which resulted in enhanced water barrier properties. This trend can be attributed to the cross-linking agents linking the polymer chains and promoting the formation of a three-dimensional network structure, which resulted in a reduction in the free volume of the polymer matrix, an increase in the tortuosity of the polymer network [15], and a reduction in the WVP value of the FK/PVA/Tris blend film.

In addition, the contents of the cross-linking agents in the composite films also influenced the WVP values. More specifically, for the TG-cross-linked blend film, a TG content of 0.05% gave a minimum WVP of 1.68 × 10^−12^ g·cm^−1^·s^−1^·Pa^−1^, which was a 45.63% drop compared with that of P-40-25. Upon increasing the TG content, a slight increase in the WVP value was observed, but this value remained lower than that of the control film. For the blend film cross-linked by CaCl_2_, the WVP decreased upon increasing the CaCl_2_ content, with 0.3% CaCl_2_ giving a minimum WVP of 1.64 × 10^−12^ g·cm^−1^·s^−1^·Pa^−1^, which is a 46.93% drop compared to that of P-40-25. Again, upon increasing the CaCl_2_ content, the WVP increased slightly. A similar trend was observed for the blend film cross-linked by genipin, with a genipin content of 0.5% giving the lowest WVP value of 2.02 × 10^−12^ g·cm^−1^·s^−1^·Pa^−1^, which is a 34.63% drop compared with that of P-40-25. This trend is consistent with the TSM data, further confirming that the optimal contents of the cross-linking agents are 0.05% for TG, 0.3% for CaCl_2_, and 0.5% for genipin.

#### 3.5.2. Oxygen Permeability (OP)

The effect of the three cross-linking agents on the OP values of P-40-25 are outlined in Table 3. As indicated, the OP values of the FK/PVA/Tris composite films increased in the presence of the cross-linking agents compared to the OP of the control film. More specifically, in the presence of 0.5%-TG, 0.5%-CaCl_2_, and 0.5%-genipin, the OP value increased from 11.78 × 10^−5^ cm^3^·m^−2^·d^−1^·Pa^−1^ for P-40-25 to 4038 × 10^−5^ cm^3^·m^−2^·d^−1^·Pa^−1^, 33,970 × 10^−5^ cm^3^·m^−2^·d^−1^·Pa^−1^, and 3531 × 10^−5^ cm^3^·m^−2^·d^−1^·Pa^−1^, respectively, indicating that the addition of a cross-linker reduced the oxygen barrier properties of the FK/PVA/Tris composite film. This trend was opposite to that observed for the WVP, as we know that the process of air permeation in a film typically involves three stages (i.e., adsorption, diffusion, and desorption). The hydrophobicity of the FK/PVA/Tris blend film surface increased with increasing the cross-linking agent contents, the adsorption of non-polar O_2_ molecules on the film surface was enhanced, and the oxygen barrier properties of the blend films were reduced. Therefore, although the addition of cross-linking agents reduced the moisture sensitivity of the composite film, they also reduced its oxygen barrier properties, with a greater influence being observed for the OP.

## 4. Conclusions

We herein reported the successful preparation of feather keratin/polyvinyl alcohol/tris(hydroxymethyl)aminomethane (FK/PVA/Tris) blend films cross-linked by transglutaminase, CaCl_2_, and genipin. Examination of the film morphology and the Fourier transform infrared data showed that the addition of cross-linking agents improved the compactness and compatibility of the FK/PVA/Tris films due to the formation of new chemical bonds, which produced a cross-linked network structure. As a result, the water vapor barrier properties and tensile strengths of the films were enhanced, while the water sensitivities and oxygen barrier properties were reduced. These properties were governed by intermolecular interactions between FK, PVA, Tris, and the cross-linking agents. Therefore, we could conclude that the use of such cross-linking agents could provide a route to improving the properties of keratin films, but the cross-linked blend films were broken into pieces during the dissolution process, which may make them meet the needs of disposable packaging in some special fields, such as fish feed packaging. The package containing the feed is put into a pond, then the packaging bag is dissolved, and the dissolved protein can be used as a nutrient supply.

## Figures and Tables

**Table 1:** Addition amount of each component in the blend films. FK: feather keratin, PVA: polyvinyl alcohol, Tris: tris(hydroxymethyl)aminomethane, TG: transglutaminase.

Sample	FK Powder (g)	6% PVA (g)	6% Tris (g)	0.1% TG (g)	0.1% CaCl_2_ (g)	0.1% Genipin (g)
P-40-25	1.2	13.33	8.33	0	0	0
0.05%-TG	1.2	13.33	8.33	1	0	0
0.1%-TG	1.2	13.33	8.33	2	0	0
0.3-TG	1.2	13.33	8.33	6	0	0
0.5%-TG	1.2	13.33	8.33	10	0	0
0.05%-CaCl_2_	1.2	13.33	8.33	0	1	0
0.1%-CaCl_2_	1.2	13.33	8.33	0	2	0
0.3%-CaCl_2_	1.2	13.33	8.33	0	6	0
0.5%-CaCl_2_	1.2	13.33	8.33	0	10	0
0.05%-genipin	1.2	13.33	8.33	0	0	1
0.1%-genipin	1.2	13.33	8.33	0	0	2
0.3%-genipin	1.2	13.33	8.33	0	0	6
0.5%-genipin	1.2	13.33	8.33	0	0	10

**Table 2:** Effect of the cross-linking agent contents on the FK secondary structure.

Sample	α-Helices (%)	β-Turns (%)	β-Sheets (%)	Random Coils (%)
P-40-25	14.25	23.91	39.33	20.36
0.05%-TG	15.61	28.64	33.68	21.26
0.1%-TG	14.85	27.60	35.27	20.74
0.3%-TG	16.19	28.05	33.55	22.38
0.5%-TG	16.17	30.66	31.40	21.83
0.05%-CaCl_2_	14.34	24.08	38.92	20.75
0.1%-CaCl_2_	15.85	27.53	33.68	21.50
0.3%-CaCl_2_	16.29	32.09	29.80	21.87
0.5%-CaCl_2_	14.37	22.07	40.37	21.38
0.05%-genipin	14.97	27.08	36.04	21.12
0.1%-genipin	14.83	26.11	37.02	20.85
0.3%-genipin	15.20	26.43	36.20	21.36
0.5%-genipin	16.21	27.78	33.62	22.74

**Table 3:** Water vapor permeability (WVP) and oxygen permeability (OP) values for the blend films.

Sample	WVP	OP
	(×10^−12^ g·cm^−1^·s^−1^·Pa^−1^)	(×10^−5^ cm^3^·m^−2^·d^−1^·Pa^−1^)
P-40-25	3.09 ± 0.1 ^f^	11.78 ± 0.65 ^a^
0.05%-TG	1.68 ± 0.08 ^a^	826.4 ± 2.53 ^d^
0.1%-TG	2.32 ± 0.09 ^cd^	820 ± 4.47 ^c^
0.3%-TG	2.46 ± 0.06 ^de^	4012 ± 2.82 ^k^
0.5%-TG	2.58 ± 0.11 ^e^	4038 ± 4.65 ^l^
0.05%-CaCl_2_	3.06 ± 0.06 ^f^	1865 ± 3.53 ^e^
0.1%-CaCl_2_	2.59 ± 0.13 ^e^	3243 ± 1.9 ^h^
0.3%-CaCl_2_	1.64 ± 0.05 ^a^	3559 ± 2.32 ^j^
0.5%-CaCl_2_	3.06 ± 0.07 ^f^	33970 ± 5.7 ^m^
0.05%-genipin	2.33 ± 0.12 ^cd^	368.3 ± 0.77 ^b^
0.1%-genipin	2.39 ± 0.1 ^cd^	2068 ± 0.12 ^f^
0.3%-genipin	2.24 ± 0.03 ^c^	2708 ± 1.45 ^g^
0.5%-genipin	2.02 ± 0.05 ^b^	3531 ± 3.61 ^i^

Note: Values are given as mean ± SD (*n* = 3). Different lowercase letters indicate significant differences (*p* < 0.05). Water vapor permeability (WVP) and oxygen permeability (OP) values for P-40-25 were from Chen et al. [23].
